# Morcellation in gynecology: short review and suggestions from Turkish Society of Minimally Invasive Gynecologic Oncology

**DOI:** 10.4274/jtgga.galenos.2020.2020.0107

**Published:** 2021-02-24

**Authors:** Salih Taşkın, Bulut Varlı, İbrahim Yalçın, Fırat Ortaç, Çağatay Taşkıran, Mete Güngör

**Affiliations:** 1 Department of Gynecologic Oncology, Ankara University Faculty of Medicine, Ankara, Turkey; 2 Department of Obstetrics and Gynecology, Ankara University Faculty of Medicine, Ankara, Turkey; 3 Department of Gynecologic Oncology, Ondokuz Mayıs University Faculty of Medicine, Samsun, Turkey; 4 Department of Gynecologic Oncology, Koç University Faculty of Medicine, İstanbul, Turkey; 5 Department of Gynecologic Oncology, Acıbadem University Faculty of Medicine, İstanbul, Turkey

**Keywords:** Myomectomy, hysterectomy, morcellation, laparoscopic surgery, vaginal surgery

## Abstract

Morcellation allows the removal of a large uterus and fibroids through small incisions with minimally invasive surgery. It helps to prevent the complications associated with large incisions in both hysterectomy and myomectomy operations. Currently, there is much debate regarding the use of power morcellation in laparoscopic hysterectomy and myomectomy, mainly due to the risk of peritoneal dissemination of undiagnosed uterine sarcomas. Unfortunately, there is no valid pre-operative diagnostic method that can differentiate sarcomas from myomas, and the currently available scientific literature regarding morcellation is insufficient. As the Turkish Society of Minimally Invasive Gynecological Oncology, we present our consensus opinion and suggestions for the preoperative evaluation and morcellation of fibroids, in line with the recent literature.

## Introduction

Morcellation is performed for reducing the size of a uterus or myoma, to ease extraction of tissues from the abdominal cavity. The history of procedure goes back to the 19th century. The first applications were mechanically made after vaginal surgery to reduce the size of the tissue (1). In this way, vaginal surgery can be also performed in when a large uterus is present, which previously generally required open surgery. In subsequent years, minimally invasive surgery started to replace most open and vaginal procedures and, as a result, the need for a new way to extract huge uteruses and myomas from smaller incisions arose.

In 1976, a laparoscopic manual morcellator, which can work through 15 mm and 10 mm incisions, was produced (2). The technical properties of subsequent equipment have improved and devices have been replaced by electromechanical morcellators which has also reduced the time required for tissue extraction (3). By 1993, the use of a morcellator with more advanced features was approved by the Food and Drug Administration (FDA) (3).

A morcellator is used when performing hysterectomy or myomectomy for large uteruses during minimally invasive surgery to avoid open surgery-related morbidities. On the other hand, if morcellation is performed in the presence of uterine malignancy, especially uterine sarcoma, which usually cannot be diagnosed preoperatively, this may cause the disease to upstage and have a negative effect on the prognosis (4,5).

In 2013, a patient was diagnosed with leiomyosarcoma after total hysterectomy, which was performed with a minimally invasive approach for a presumed benign uterine fibroid and in the later staging surgery, intraperitoneal spread was observed. After this case was published, a debate started about morcellator usage and in 2014 the FDA discouraged the use of laparoscopic power morcellation during hysterectomy or myomectomy for uterine fibroids (6). In November 2014, the FDA updated its recommendations and specified contraindications for morcellation (7):

1. Morcellators are contraindicated for removal of uterine tissue containing suspected fibroids in patients who are peri- or post-menopausal or are candidates for en-bloc tissue removal through the vagina or mini-laparotomy incision.

2. Morcellators are contraindicated in patients with uterine fibroids suspicious for malignancy.

However, the scientific basis of this advice was not clear and definition of perimenopause was not explained. Despite the FDA’s clear advice against morcellation, some national societies have not made a strict recommendation to prohibit morcellation (8,9,10,11,12).

The Turkish Minimally Invasive Gynecologic Oncology Society formed a working group on this subject and prepared suggestions in the light of current literature, which will guide both surgeons and patients.

## Uterine sarcoma types and occult sarcoma risk in presumed myoma

Endometrial adenocarcinoma constitutes nearly 95% of all uterine malignant tumors (13). Mostly, diagnosis is obtained through pre-operative endometrial sampling and it is rarely diagnosed incidentally after hysterectomy. However, pre-operative diagnosis of uterine sarcomas, which make up 5% of uterine tumors, is not possible most of the time (13). These patients are at highest risk from inappropriate morcellation.

Leiomyosarcoma, endometrial stromal sarcoma and rhabdomyosarcoma are most common types of uterine sarcomas. Unfortunately, the exact rate of post-operative sarcoma diagnosis is not known in patients who are presumed to have benign fibroids pre-operatively. Since this is a rare situation, most of the relevant studies are retrospective and contain much bias. In a report of the FDA, the incidence of all sarcomas and leiomyosarcomas were reported as 1/350 and 1/458, respectively (6,7).

However, it is also seen that this rate varies according to the method of studies. In a meta-analysis of 133 studies (14), occult leiomyosarcoma risk was calculated as 1/1960 when both retrospective and prospective studies were included, but this rate dropped to 1/8300 when only prospective studies were considered. In studies investigating the incidence of sarcoma in patients who underwent morcellation during myomectomy or hysterectomy for presumed benign disorders, the highest rate was reported as 0.6% (15). Recently, two studies from Turkey reported the incidence of occult uterine sarcomas (16,17). Topdagi Yilmaz et al. (16) reported the incidence of unexpected uterine sarcoma in patients who underwent hysterectomy for benign indications as 0.6% (7/1050). In addition, Yorgancı et al. (17) investigated the rate of occult uterine sarcoma in 18,604 women who underwent hysterectomy or myomectomy with a pre-operative diagnosis of uterine leiomyoma and occult uterine sarcoma incidence was 0.3% (56/18604).

## Possible adverse effects of morcellation: sarcoma and benign conditions

Morcellation can be performed manually, either using scissors or scalpel, or power morcellation can be performed using electromechanical devices. It can be performed during minimally invasive surgery or vaginal surgery. The procedure can be performed un-contained, contained (in bag) or using a mini-laparotomic incision.

After hysterotomy, regardless of morcellation, malignant cells, if present, may spread to the peritoneal cavity. During morcellation, the specimen is divided into smaller pieces in the peritoneal cavity and, irrespective of the malignancy potential, some problems may arise including spread of tissues into the peritoneal cavity, incomplete removal of tissue fragments, and microscopic residues becoming peritoneal implants. Thus, an increased incidence of benign peritoneal diseases, including parasitic leiomyoma, endometriosis and extensive intraperitoneal leiomyomatosis, have also been reported after morcellation (18). It should be kept in mind that morcellation significantly increases the risk of these benign sequelae compared to the risk of spreading malignancies.

Long-term survival is not favorable in patients with leiomyosarcoma (19). Besides, there are publications supporting the idea that morcellation can worsen the stage and negatively affect survival in the presence of malignancy (5,20,21). In a study evaluating the effect of morcellation on survival, in the “no morcellation” group, 1-year mortality rate was 5.3% and in the morcellation group this rate was 18.2% (20). Patients who were diagnosed with stage 1 sarcoma or smooth muscle tumors of uncertain malignant potential during initial surgery were operated after a median of 33 days (22) and widespread peritoneal disease was found in 28% and 25% of the patients, respectively (22). Although the studies are retrospective, it was found that hysterotomy affects survival negatively compared to intact hysterectomy. In morcellated sarcoma cases, the risk of abdominopelvic spread increased significantly (44% vs 12.9%) and survival decreased significantly compared to those in whom morcellation was not performed (5). In another case series, the 1-year mortality rate was found to be significantly higher in the morcellation group (20). Result of a meta-analysis also supported increased risk of recurrence and death (21).

After morcellation, integrity of the specimen is damaged and this can cause both difficulty in pathological examination and it may also adversely affect the diagnosis and staging procedures (23).

## In-bag morcellation

In order to prevent or reduce the adverse effects of intraperitoneal uncontained morcellation, morcellation in a closed peritoneal space has been suggested as a possible solution. The most popular method is in-bag morcellation but its potential for avoiding harmful effects and superiority over other morcellation strategies is unconfirmed and needs to be studied further. In addition, there is no consensus opinion from interested societies that in-bag morcellation will prevent morcellation related complications.

In studies evaluating a limited number of patients, tissue or dye leakage or spreading out of the bag were observed in 9-33% of the cases when morcellation was performed in the bag (24,25). Some of the leaks, perhaps, represent microscopic spread. However, there is no data yet on whether this will affect survival in case of malignancy.

## Preoperative sarcoma diagnosis

Risk factors include age, history of pelvic irradiation, tamoxifen usage, genetic syndromes (i.e. hereditary leiomyomatosis and renal cell cancer mutation, Lynch syndrome) and history of retinoblastoma in childhood and have been shown to increase the risk of sarcoma (9). If the lesion shows rapid growth in a 3-month period (exact clinical and radiological criteria have not been determined), and especially if there is a lesion greater than 8 cm in the menopausal period, or lesions with central necrosis, heterogeneous appearance, non-calcified cystic degeneration and irregular high blood supply may arouse suspicion of sarcoma (9). However, none of these criteria is effective enough to establish a definitive preoperative diagnosis (26).

## Preoperative endometrial biopsy

Although it is an effective diagnostic method in the diagnosis of endometrial pathologies, the effectiveness of endometrial biopsy in the diagnosis of uterine sarcomas is low. It was shown that endometrial biopsy identified only 36% of leiomyosarcomas in submucous lesions (27). In the diagnosis of endometrial stromal sarcoma, the sensitivity was 33% (28). Localization of lesions can vary significantly, and therefore endometrial biopsy is not considered as a useful preoperative diagnostic test in these lesions. Besides, in asymptomatic women, no benefit has been shown. However, endometrial biopsy can help clinicians in patients with preoperative abnormal uterine bleeding.

## Imaging methods

Ultrasonography is the first and most frequently used radiological method but differential diagnosis between leiomyoma and sarcoma cannot be made always (29,30). In color doppler, atypical vessel pattern, low resistance index and high systolic velocity is observed in sarcomas (31). However, depending on variables, such as location of the lesion, menopausal status of the patient, and size of the lesion, ultrasonographic features of sarcoma and leiomyomas may overlap and are not distinctive in the majority of cases.

Magnetic resonance imaging (MRI) may show more diagnostic accuracy in differentiation of leiomyoma and sarcoma (13,32). Features such as necrosis, rapid growth, intense contrast enhancement, and restriction on diffusion-weighted imaging can ease the diagnosis and help to differentiate sarcomas from leiomyomas. However, specificity and positive predictive value are low (32). If diffusion-weighted images and contrast imaging are used, discrimination can increase (33). However, despite studies that reported diagnostic efficacy as 88% with these methods (34), some studies have reported imprecision in the successful distinction of fibroid and sarcoma (33). Therefore, the role of MRI should be evaluated in further studies involving more patients. Also, MRI is not recommended for routine use in all lesions and should be used after ultrasonography, in the presence of clinical suspicion (13).

Computed tomography and a positron emission tomography scan are not helpful to discriminate leiomyoma and sarcoma, and they should not be used pre-operatively, solely for this purpose.

## Biochemical markers

It is thought that elevated levels of lactate dehydrogenase (LDH) may serve as an indicator of necrosis in the tumoral tissue and invasion into the intravascular area in the presence of sarcoma. Studies have shown that increased levels of LDH are significantly more frequent in the sarcoma group than in the leiomyoma group (35). In one study, the diagnostic success rate of a combination of LDH and MRI was reported to be 100% (32). Success in evaluations with LDH subtypes was also reported, investigating LDH isozyme type 1 and 3 (32,35). In a study using receiver operating characteristic curve analysis for the prediction of sarcoma in the pre-operative period, the optimum cut-off value for LDH was 279.0 U/L (36). However, further studies are needed to confirm the utility of LDH in differential diagnosis.

## Intraoperative management

Some characteristics of uterine lesions raise suspicion for sarcoma intraoperatively. These are: no clear mass borders like leiomyoma; no bulging during uterine incision; soft, homogenous, yellowish appearance; and increased tissue fragility. However, these features may also present in patients with degenerative myoma or after use of pre-operative hormonal treatment. In advanced stages, sarcomas may lead to overgrowth and local invasion to adjacent organs (e.g bladder, rectum). Intraoperative frozen-section analysis does not have much efficacy and diagnostic accuracy was reported as only 11-38% (37).

## Postoperative management

A uniform clinical management plan for patients with morcellated uterine sarcoma does not exist. Several authors have advised completion of surgery with hysterectomy in case of myomectomy and the abdominal cavity can be evaluated for the presence of metastatic implants (38). Also, patients with late surgical (>30 days) re-exploration had a higher mortality rate (39).

## Opinions and suggestions

Since uterine sarcomas are rare and most of the available data are based on retrospective studies, it is difficult to provide certain and conclusive suggestions. The following opinions and suggestions are presented in line with the available data. The following statements can be potentially modified or altered as per new evidence.

1. There is no method that can definitively differentiate sarcomas pre-operatively in patients who are going to be operated with a preliminary diagnosis of uterine myoma.

2. Uterine sarcomas usually occur in women of advanced age, but there is no exact age limit. Especially in patients aged >35 years who are being considered for morcellation, it is recommended that the risk factors should be investigated, that the patient should be examined with advanced imaging methods in case of suspicion, and the necessary precautions should be taken to prevent peritoneal contamination in case of intraoperative suspicion.

3. Ultrasonography is the recommended first-line imaging method. Routine MRI is not recommended for every pre-operative patient and should be performed when malignancy is suspected. A pre-operative endometrial biopsy may only be useful in patients with abnormal uterine bleeding. Its effectiveness in diagnosing sarcomas is very poor.

4. Survival outcomes are worse in uterine sarcomas, even in the early stages, compared to endometrial malignancies. The morcellation of sarcomas can result in disease progression and worsen survival outcomes compared to non-morcellation.

5. The peritoneal seeding resulting from morcellation increases the incidence of benign sequelae. These sequelae account for the vast majority of morcellation-related morbidities and should not be ignored.

6. Although it is assumed that morcellation with tissue containment may be protective against negative outcomes, there is not enough evidence regarding the preventive efficacy of this method. Further studies are needed to establish conclusive data.

7. Patients should be informed in detail regarding the advantages of minimally invasive surgery and the risks of morcellation. In patients in whom malignancy is suspected, morcellation should be avoided (or not performed at all), regardless of the patient’s consent.

8. In patients who will be operated with a preliminary diagnosis of uterine myoma, intact removal of the uterus may be primarily considered, depending on the patients’ fertility preferences and age.

9. Studies should be designed to determine the efficacy of pre-operative diagnostic methods and the preventive potential of contained morcellation techniques. These studies should aim for the inclusion of as many centers as possible due to the low prevalence of the disease.
